# Genome instability model of metastatic neuroblastoma tumorigenesis by a dictionary learning algorithm

**DOI:** 10.1186/s12920-015-0132-y

**Published:** 2015-09-10

**Authors:** Salvatore Masecchia, Simona Coco, Annalisa Barla, Alessandro Verri, Gian Paolo Tonini

**Affiliations:** DIBRIS, Università degli Studi di Genova, Genova, Italy; Lung Cancer Unit; IRCCS A.O.U. San Martino – IST, Genova, Italy; Neuroblastoma Laboratory, Onco/Hematology Laboratory, Department of Woman and Child Health, University of Padua, Pediatric Research Institute, Fondazione Città della Speranza, Padua, Corso Stati Uniti, 4, 35127 Padua, Italy

## Abstract

**Background:**

Metastatic neuroblastoma (NB) occurs in pediatric patients as stage 4S or stage 4 and it is characterized by heterogeneous clinical behavior associated with diverse genotypes. Tumors of stage 4 contain several structural copy number aberrations (CNAs) rarely found in stage 4S. To date, the NB tumorigenesis is not still elucidated, although it is evident that genomic instability plays a critical role in the genesis of the tumor. Here we propose a mathematical approach to decipher genomic data and we provide a new model of NB metastatic tumorigenesis.

**Method:**

We elucidate NB tumorigenesis using Enhanced Fused Lasso Latent Feature Model (E-FLLat) modeling the array comparative chromosome hybridization (aCGH) data of 190 metastatic NBs (63 stage 4S and 127 stage 4). This model for aCGH segmentation, based on the minimization of functional dictionary learning (DL), combines several penalties tailored to the specificities of aCGH data. In DL, the original signal is approximated by a linear weighted combination of *atoms*: the elements of the learned *dictionary*.

**Results:**

The hierarchical structures for stage 4S shows at the first level of the oncogenetic tree several whole chromosome gains except to the unbalanced gains of 17q, 2p and 2q. Conversely, the high CNA complexity found in stage 4 tumors, requires two different trees. Both stage 4 oncogenetic trees are marked diverged, up to five sublevels and the 17q gain is the most common event at the first level (2/3 nodes). Moreover the 11q deletion, one of the major unfavorable marker of disease progression, occurs before 3p loss indicating that critical chromosome aberrations appear at early stages of tumorigenesis. Finally, we also observed a significant (*p =* 0.025) association between patient age and chromosome loss in stage 4 cases.

**Conclusion:**

These results led us to propose a genome instability progressive model in which NB cells initiate with a DNA synthesis uncoupled from cell division, that leads to stage 4S tumors, primarily characterized by numerical aberrations, or stage 4 tumors with high levels of genome instability resulting in complex chromosome rearrangements associated with high tumor aggressiveness and rapid disease progression.

**Electronic supplementary material:**

The online version of this article (doi:10.1186/s12920-015-0132-y) contains supplementary material, which is available to authorized users.

## Background

Neuroblastoma (NB) is a clinically and biologically heterogeneous pediatric cancer, the onset of which can be localized or disseminated disease. Disseminated tumors are classified as clinical stages 4S and 4. Stage 4S occurs in infants, usually have a good prognosis without any treatments, although a small fraction of stage 4S patients can have a disease progression requiring chemotherapy [1]. Both infants and children can present stage 4 NB, but older patients have usually a worse outcome with a rapid disease progression that leads to death in more than half of patients [2].

Genome-wide studies have showed that gains of chromosomes 2p, 7 and 17q are frequently present together with losses of 1p, 4p, 9p, 11q, and 14q in stage 4 tumors, whereas stage 4S tumors frequently display numerical copy number aberrations (CNAs) [3–5]. The origin of such complex chromosomal aberrations is still unclear, and there are currently no accurate models of NB tumorigenesis. Nonetheless, experimental evidence indicates that NB is characterized by a high level of genome instability [6] and that CNAs accumulate in an age-dependent manner [7, 8].

**Fig. 1:**
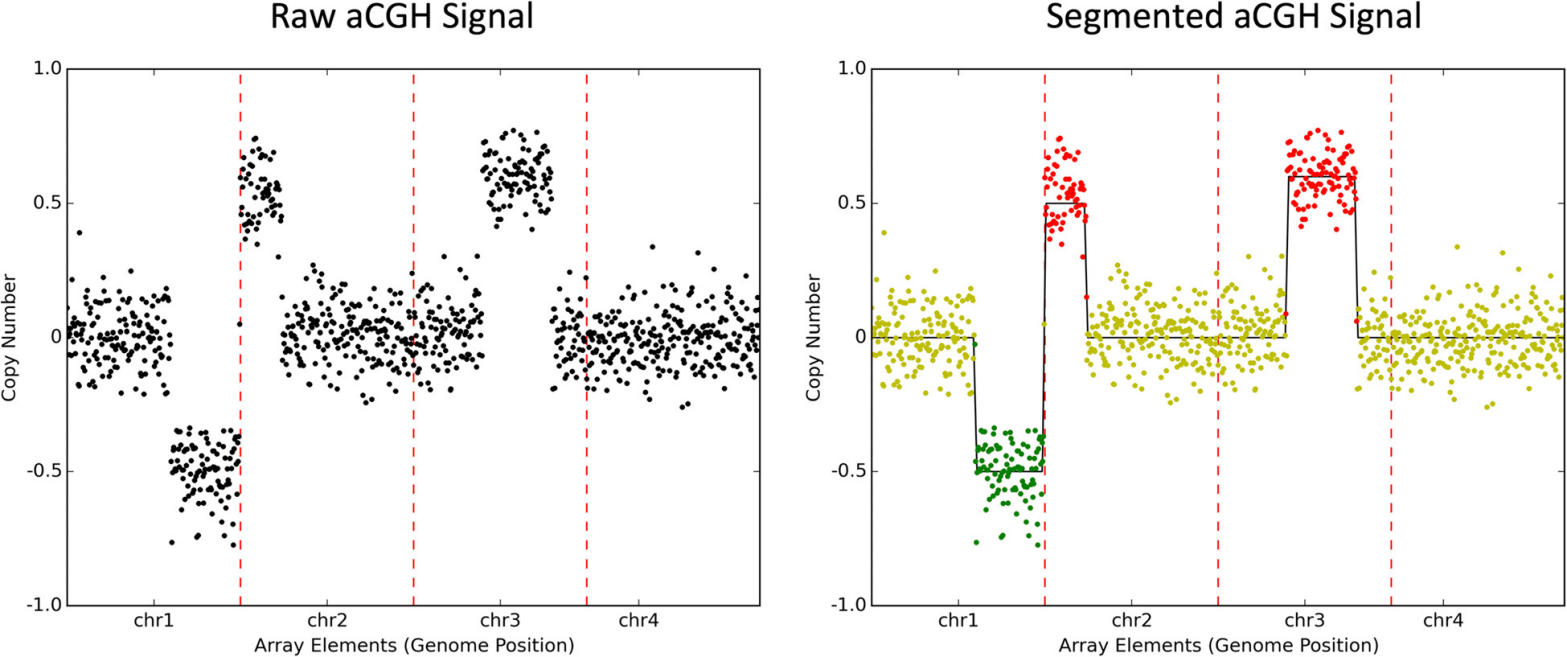
An aCGH signal before and after segmentation. An aCGH profile can be thought of as the concatenation of the log-ratio values ordered by chromosomes and by chromosomal location. In the top plot, each black dot corresponds to a probe placed at a given chromosomal location (x-axis) and with a corresponding estimated a log-ratio of the CNAs for the hybridized control and patient (y-axis). Probes are sorted according to their chromosomal location, as example, from chromosome 1 to chromosome 4 and each dotted red line represents boundaries among chromosomes. The bottom plot shows the same aCGH profile after segmentation. The thin black line is a piecewise constant signal obtained as a result of the segmentation. The red dots indicate *gain* whereas the green dots correspond to probes where a *loss* occurred

As a result of the publicly available high-throughput array comparative genomic hybridization (aCGH) data repositories in the Gene Expression Omnibus (GEO), it is now possible to investigate CNAs in large cohorts of NB samples. A signal measured with the aCGH technology is made of a piecewise constant component plus some composite noise. The typical analysis on such data is segmentation, which is the automatic detection of chromosome loci where CNAs (amplifications or deletions) occur, as shown in the Fig. 1. Beyond that, it is crucial to understand how these alterations co-occur. This turns to identifying shared patterns (latent features) in the data, which may reveal a genotype-phenotype relationship.

Several methods have been suggested for the extraction of CNAs based on different principles, such as filtering (or smoothing), segmentation, breakpoint-detection and calling [9–16], taking into account one sample at a time [17].

Especially in cancer diseases, where mutations happen very frequently, joint-analysis of aCGH samples could be helpful to filter out unshared mutations among (at least a subset of) samples. One of the first works applying this approach was performed by Pique-Regi et al. [18] where the authors extended their previous model [13] to the “multi-sample” analysis. Following this scheme, many other approaches were proposed usually extending the “one-by-one” criterion to a “multi-sample” application [19–21] and in some cases extending this approach also to the joint-normalization of data [22].

Moreover, some interesting recent results were obtained by adopting statistical learning methods based on regularization for a joint segmentation of many aCGH profiles at once. Previous results [11, 23–25] obtained following this method are based on total variation (*TV*) or fused lasso signal approximation.

In this context, we use E-FLLat (Enhanced Fused Lasso Latent Feature Model) [26], a novel model for aCGH segmentation, based on the minimization of functional dictionary learning (DL) combining several penalties tailored to the specificities of the data at hand. In DL, the original signal (*i.e.*, the aCGH sample) is approximated by a linear weighted combination of the *atoms* (*i.e.*, a set of elementary alterations), which are the elements of a learned *dictionary*.

**Fig. 2:**
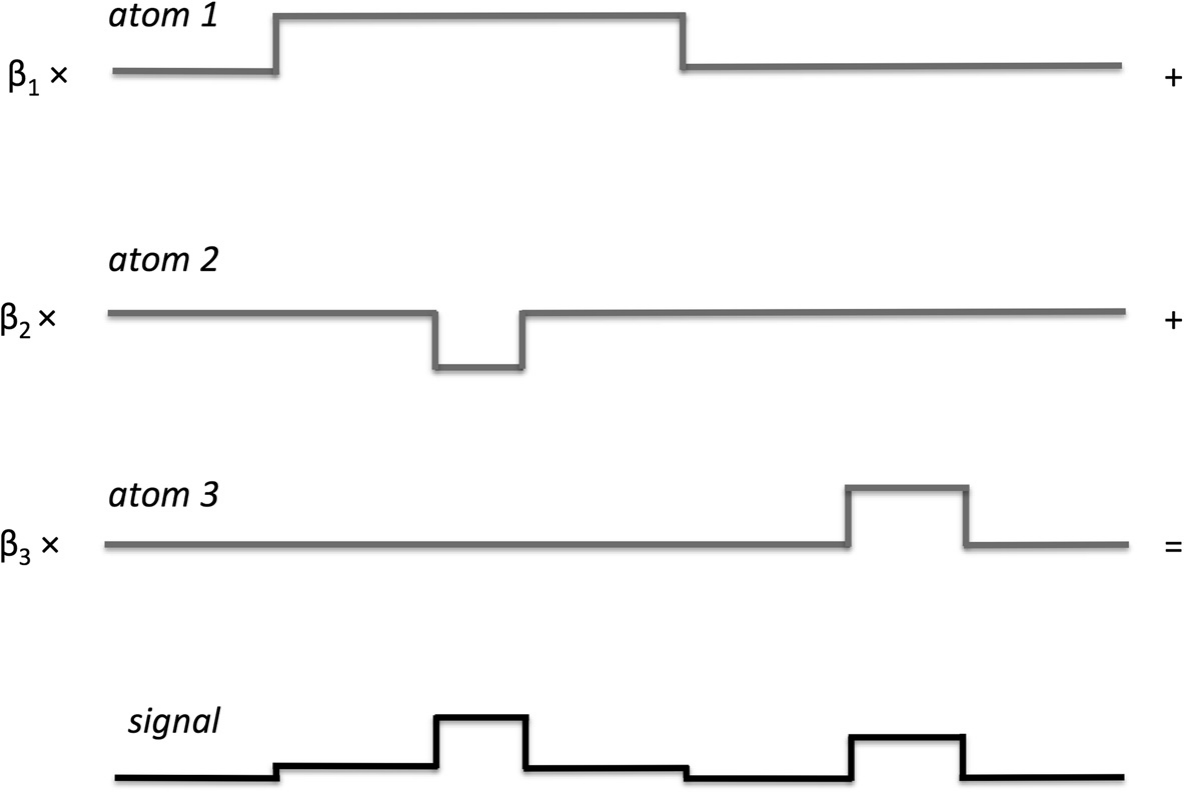
A piecewise constant signal as weighted linear combination of atoms. The piecewise constant signal at the bottom of the figure can be obtained by linearly combining a set of three elementary alterations (atoms). Each atom is multiplied by a weighting factor (coefficient) β and then added up to obtain the final signal

We assumed that each sample can be approximated by a weighted combination of some of the identified atoms. A simple example of this concept is shown in the Fig. 2 where the signal can be obtained as the weighted sum of three elementary alterations (atoms). In the analysis of the NB data, we first identified the atoms using E-FLLat; then, we applied an inference method [27] to place the atoms in a set of hierarchical structures (trees) that may shed light on NB oncogenesis.

Thus, we used the aCGH data and a statistical inference method based on dictionary learning to propose a Genome Instability Progressive (GIP) model of tumorigenesis for metastatic NB, which has one of the most aggressive known pediatric cancer phenotypes.

## Methods

### Data description

We analyzed six publicly available datasets from the GEO [28] for E-FLLat analysis (Table 1). We used a variation of the alignment algorithm of [29] consisting of three steps: 1) mapping to the reference genome, 2) signal smoothing for noise reduction, and 3) alignment.

**Table 1:** Description of aCGH data collected from public available datasets

^a^Platform	^b^GEO code	^c^GEO serie	^d^NB stage 4S	^d^NB stage 4	^e^Total samples
AgilentCgh2x105k	GPL4093	GSE25771	16	7	23
AgilentCgh4x44k	GPL2873	GSE25771 GSE35953	10	39	49
AgilentCgh4x44k	GPL2879	GSE25771	5	1	6
AgilentCgh4x44k	GPL5477	GSE14109 GSE25771 GSE35953	17	73	90
AgilentCgh4x44k	GPL11633	GSE26494	1	0	1
Nimblegen4x72k	GPL8971	GSE26494	14	7	21
^e^Total Samples			63	127	190

^a^Platform type, ^b^GPL file code from GEO, ^c^GEO series number, ^d^number of Stage 4 and 4S neuroblastoma samples, ^e^samples number used in the analysis

### Data alignment and normalization

1. *Mapping of the Human Genome (HG19):* First, we proceeded by mapping the probe sets on the HG19 [30]. The Agilent samples were mapped using mapping files from UCSC (44 k and 105 k)^1^, whereas the Nimblegen data were mapped using the lift-over function available at UCSC^2^**.**
2. *Normalization:* For the Agilent platforms, we first performed a check of the quality control (QC) results, discarding those probes associated with a poor QC value. For the normalization of all of the data, we used CGHnormaliter [31]. Each input file (sample) has a corresponding normalized output containing information on the call, segmentation and normalized log-ratio of all 22 autosomes. As the output, the algorithm provides the CNAs estimated for each unique probe set on the chip.
3. *Alignment:* As opposed to Jong et al. [29], who sampled each chromosome N times, we decided to sample the chromosomal bands, excluding the non-coding short arms 13p, 14p, 15p and 22p and the sexual chromosomes. Each of the resulting 795 chromosomal bands was sampled *N =* 10 times. For each of the new virtual 7950 probes, we performed a K-NN procedure that assigns an estimated expression to the new virtual probe by considering K = 10 nearest neighbors all belonging to the same chromosomal band. K = 10 was the upper bound; therefore, we used ten values at most to estimate the assigned expression. Finally, also in contrast to Jong et al. [29], we excluded the z-score transformation, due to its drastic effect on the signal mean. After the alignment and normalization, the dataset was composed of 190 samples represented by 7950 probes. The chromosomal bands contained 10 probes each.

### E-FLLat: a dictionary learning-based approach for aCGH segmentation

E-FLLat is a dictionary learning-based model for aCGH segmentation [23]. We are given *S* samples ${\left({\mathrm{y}}_S\right)}_{\mathit{\mathsf{1}}\mathit{\le}\mathit{\mathsf{S}}\mathit{\le}\mathit{\mathsf{S}}}$, with *y*_*s*_ ∈ ℝ^*L*^. The aim of dictionary learning is to seek *J simple* atoms (***β***_*j*_)_1 ≤ *j* ≤ *J*_ with *β*_*j*_ ∈ ℝ^*L*^, which may provide a complete representation of all of the samples in the sense that

$$ {\boldsymbol{y}}_{\boldsymbol{s}} \cong {\displaystyle {\sum}_{\boldsymbol{j}=1}^{\boldsymbol{J}}}{\boldsymbol{\theta}}_{\boldsymbol{j}\boldsymbol{s}}{\boldsymbol{\beta}}_{\boldsymbol{j}}\kern0.75em \forall \boldsymbol{s}=1, \dots,\ \boldsymbol{S} $$

for some vectors of coefficients ***θ***_***s***_ = (***θ***_***js***_)_1 <*j* <*J*_

Thus, the E-FLLat model is as follows:

$$ \begin{array}{c}\hfill \underset{{\boldsymbol{\theta}}_{\boldsymbol{s},}{\boldsymbol{\beta}}_{\boldsymbol{j}}}{ \min }{\displaystyle \sum_{\boldsymbol{s}=1}^{\boldsymbol{S}}}{\boldsymbol{y}}_{\boldsymbol{s}}-{\displaystyle \sum_{\boldsymbol{j}=1}^{\boldsymbol{J}}}{\boldsymbol{\theta}}_{\boldsymbol{j}\boldsymbol{s}}{{\boldsymbol{\beta}}_{\boldsymbol{j}}}^2 + \boldsymbol{\lambda} {\displaystyle \sum_{\boldsymbol{j}=1}^{\boldsymbol{J}}}{{\boldsymbol{\beta}}_{\boldsymbol{j}}}_1^2+\boldsymbol{\mu} {\displaystyle \sum_{\boldsymbol{j}=1}^{\boldsymbol{J}}}\boldsymbol{T}{\boldsymbol{V}}_{\boldsymbol{w}}\left({\boldsymbol{\beta}}_{\boldsymbol{j}}\right) + \boldsymbol{\tau} {\displaystyle \sum_{\boldsymbol{s}=1}^{\boldsymbol{S}}}{{\boldsymbol{\theta}}_{\boldsymbol{s}}}_1^2\hfill \\ {}\hfill \boldsymbol{s}.\boldsymbol{t}.\ 0\le {\boldsymbol{\theta}}_{\boldsymbol{j}\boldsymbol{s}}\le {\boldsymbol{\theta}}_{\boldsymbol{max}}\kern0.75em \forall \boldsymbol{j}=1, \dots,\ \boldsymbol{J}\ \forall \boldsymbol{s}=1, \dots,\ \boldsymbol{S}\kern2em \hfill \end{array} $$

The *weighted total variation*$\boldsymbol{T}{\boldsymbol{V}}_{\boldsymbol{w}}\left({\boldsymbol{\beta}}_{\boldsymbol{j}}\right) = {\displaystyle {\sum}_{\boldsymbol{l}=1}^{\boldsymbol{L}-1}}{w}_{\boldsymbol{l}}\left|{\boldsymbol{\beta}}_{\boldsymbol{l}+1,\boldsymbol{j}}-{\boldsymbol{\beta}}_{\boldsymbol{l},\boldsymbol{j}}\right|$ is a generalized total variation due to the presence of the weights ***w*** = (*w*_*l*_)_1 ≤ *l* ≤ *L* − 1_ ∈ ℝ^*L* − 1^. This modification is introduced to relax the constraint of *small jumps* on the atoms at some points. In fact, we imposed *w*_*l*_ = 0 at the boundaries between the chromosomes and at the chromosome centromeres. Elsewhere, *w*_*l*_ was set according to a position-dependent weighting schema as in [32].

### Post-processing and dictionary interpretation

After the segmentation process, the dictionary was post-processed to set a level of detail that was sufficiently general for the subsequent step of investigation. If one probe was detected as altered by E-FLLat, the smallest chromosomal band that contains that probe was considered as altered. Then, alterations occurring on adjacent chromosomal bands were merged and considered as one alteration occurring on the merged band.

### Carcinogenesis tree reconstruction

Once the E-FLLat approach identified the atoms, we used MTreeMix [27], a software package for learning and using mixture models of oncogenic trees, to describe evolutionary processes that are characterized by the ordered accumulation of permanent genetic changes. A tree is a hierarchical structure with one root node and a well-ordered set of nodes. The elements composing the tree are nodes and links. The depth of a node is the distance in links from the root node. The *n*-th level is defined as the set of nodes with distance *n* from the root node.

## Results

E-FLLat provides a new representation of the data in terms of a set of atoms (dictionary) and matrix of coefficients ϴ. Each sample can be approximated by a sparse linear combination of the atoms weighted by its corresponding set of coefficients (columns of the ϴ matrix). Each atom is a unique element of the learned dictionary and represents an elementary pattern of highly correlated alterations that co-occur in the dataset. We separately applied E-FLLat to the stage 4S and 4 subsets, represented by two matrices (63x7950-dimensional and 127x7950-dimensional, respectively).

The atoms for stage 4S and 4 are listed in Table 2. Each atom is the set of relevant CNAs selected by E-FLLat and post-processed as described above. The number of atoms was chosen according to a principal component analysis (PCA) analysis (see Additional file 1 Figure S1) and was applied separately for stage 4S and stage 4 data matrices. The PCA showed that 90 % of the covariance of stage 4S samples can be explained using J = 19 atoms, whereas J = 42 atoms are required for stage 4 samples. Therefore, we chose J = 16, which is sufficient to explain at least 70 % of the data covariance in both cases.

**Table 2:** Atoms characterizing Stage 4S and stage 4 samples

	Stage 4 s	Stage 4
Atom	Chromosome bands	Chromosome bands
A1	+2, +12	+2p22.3, +2p23
A2	+7, +17	+17q
A3	+6, +13	+2p24.3, +7q, +17q
A4	−21q, −4p	+2p24, −1p
A5	−14q	+2p2, +12q
A6	−8p	+17p, +17q1
A7	+2p2, +7	+18
A8	−14q2	+2p
A9	−14q	−3p
A10	+2p1, +2q, +7	+17p
A11	−4, −10	+7
A12^a^	--	+12
A13	−3	+2p2
A14^a^	--	--
A15	+2	−11q
A16^a^	+12, +17q	--

^a^Atom 12 is not associated with any relevant alteration for stage 4S,

A14 for both 4S and 4, A16 for stage 4

**Fig. 3:**
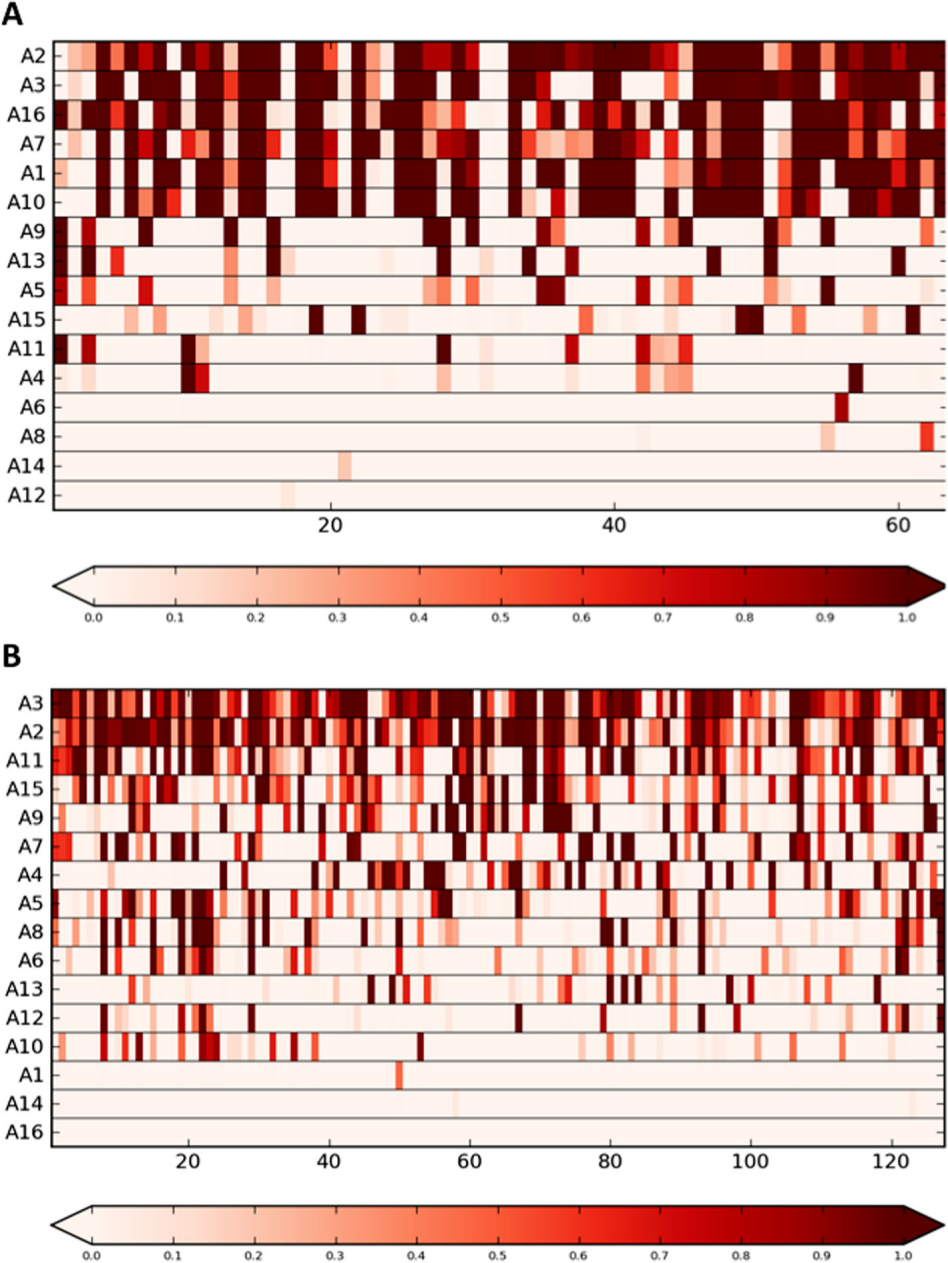
Representation of Θ coefficients for stage 4S and stage 4 tumors. The stage 4S (**a**) and stage 4 (**b**) tumors are reported in the columns, whereas the atoms are in the rows. Each sample is approximated by a linear combination of atoms weighted by the Θ coefficients. The atoms in the Θ matrix are sorted according to their use in the sample representation, *i.e.,* the most used atoms are in the top rows. The coefficients range from 0 to 1, as indicated by the underlying color bar, and darker hues correspond to higher coefficient values

Figure 3 shows the representation coefficients (ϴ matrix) for stage 4S and stage 4 samples, whose atoms are listed in Table 2. As expected, the atoms identified by E-FLLat highlight that stage 4S samples are characterized by numerical alterations (A1, A2, A3, A11, A13, A15), whereas the stage 4 data mainly show segmental aberrations. The only numerical alterations in stage 4 tumors affected chromosomes 18 and 7 (atoms A7 and A11).

After the identification of the relevant atoms, the trees were inferred using the MTreeMix algorithm. Each root node in the trees is associated with the portion of patterns described by the corresponding tree.

**Fig. 4:**
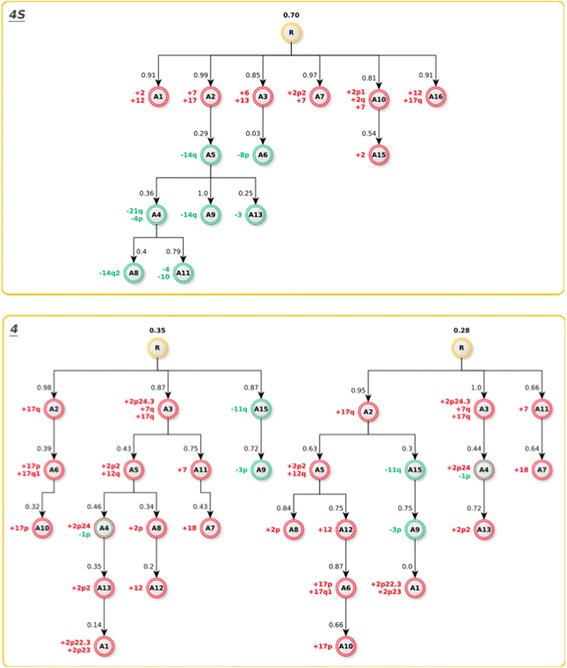
Oncogenetic Trees. The reconstructed atom tree for stage 4S (top) shows several initial events of which only one with sublevels. Conversely, both stage 4 reconstructed atom trees (bottom) are marked branched (up to five sublevels) The root node R (yellow) is associated with a weight corresponding to the portion of mutation patterns represented by the tree. The missing portion is associated with the random mutations tree (data not shown). The edges are weighted according to the frequency of the corresponding mutation occurrence. Node color codes: the red node is associated with chromosome gain, the green node is associated with chromosome loss, and the green and red node indicates co-occurring loss and gain

The hierarchical structures for stage 4S and 4 tumors are depicted in Fig. 4. The stage 4S tree shows six events (atoms) with probabilities that range from 0.81 to 0.99, suggesting that they occur in most of the represented samples. As expected at the first level the atoms are characterized mainly by whole chromosome gains except to the unbalanced gains at 17q and 2p or 2q.

Conversely, the high CNA complexity found in stage 4 tumors, requires two different trees to be properly represented. Notably each tree shows three atoms only at the first level, where the unbalance gain of 17q is the most common aberration occurring in two out of three nodes.

## Discussion

Metastatic stage 4S and stage 4 NB tumors are characterized by distinct genome profiles and clinical behavior. In particular, the majority of stage 4 NB display several structural CNAs that confer marked aggressiveness to these tumors [5]. How these chromosome aberrations are originated and how lead to transformation and carcinogenesis is still unclear, although experimental evidence indicates that genomic instability can play a critical role in the genesis of this tumor [7, 8].

Here, we propose the GIP model of carcinogenesis for metastatic NBs. To create this model, we used E-FLLat, a dictionary learning-based method that naturally groups the most relevant alterations in elementary patterns and sorts them according to how many times such patterns occurred in the data. This behavior is a built-in property of dictionary learning approaches, and in addition to being an advantage *per se* by providing a compact way of analyzing the coexisting alterations; it also eases the computational burden of the subsequent tree inference process. We used three different penalties that allowed us to segment the signal incorporating the characteristics of the aCGH data. Indeed, an aCGH signal is a stepwise constant, and alterations may co-occur in different chromosomes at the same time. We describe the E-FLLat model, modifying some notations and illustrating the rationale behind the chosen constraints. When dealing with aCGH, ***TV***_***w***_ allows the treatment of signals generated by several chromosomes as a whole while still guaranteeing an independent analysis for each chromosome, ensuring the capability of identifying concomitant alterations occurring on different chromosomes. In the E-FLLat model, coefficients are constrained to be positive and are bounded by *θ*_*max*_ = 1. This constraint prevents a cancellation effect in the representation of the signal, leading to a simpler matrix of coefficients and a matrix of atoms, which more clearly reveal the latent patterns in the data. In this way, the interpretability of the results is improved. For example, when losses and gains occur within data at the same locus, the model selects different atoms to describe them as different phenomena. The coefficients are further penalized by the term $\boldsymbol{\tau} {\displaystyle {\sum}_{\boldsymbol{s}=1}^{\boldsymbol{S}}}{{\boldsymbol{\theta}}_{\boldsymbol{s}}}_1^2$, which induces sparsity in the set of weights associated with each sample separately. This feature permitted the model to regulate how many different atoms of each sample can be combined to reconstruct the original signal. Then, we used the term ${\displaystyle {\sum}_{\boldsymbol{j}=1}^{\boldsymbol{J}}}{{\boldsymbol{\beta}}_{\boldsymbol{j}}}_1^2$, which induces a structured sparsity in the columns of the matrix of the atoms. Only inexact algorithms can solve the minimization of the model in the E-FLLat function. In our implementation, we use an alternating proximal algorithm [26], which provides an approximation of the exact solution with a controlled error. The choice of the regularization parameters (λ, μ, τ) is determined according to the Bayesian Information Criterion [33] that mitigates the problem of over-fitting by introducing a penalty term for the complexity of the model. The choice of the number of atoms J is made with a criterion based on PCA [34]. PCA seeks to optimally represent the data in terms of the minimal reconstruction error, *i.e.,* the mean-square-error between the representation and the original data, hence projecting onto the first eigenvectors of the covariance matrix of the inputs. We apply PCA to the data matrix, identifying the minimum number of eigenvectors necessary to explain at least 70 % of the data covariance. We chose this value as the number of atoms J. Indeed, all of the state-of-the art tree inference methods [27, 35] have prohibitively long lists of single alterations, which is a very common scenario when dealing with complex diseases such as NB tumors.

The idea is to establish a mixture model M of K trees T_*k*_ with a maximum likelihood approach (22). In MTreeMix, the estimation of a single tree is based on solving a maximum weight branching problem by a combinatorial algorithm. The mixture model is fitted iteratively with an EM algorithm: in the E-step, the algorithm assigns the data to the tree components and estimates the missing data; in the M-step, it fits the trees on the respective subsets. As input, MTreeMix considers the list of events or atom patterns, *i.e.,* the lists of atoms used to reconstruct each sample. An event is an entry of a binary J-dimensional vector displaying which atoms are used by E-FLLat to reconstruct the corresponding sample (entries 0 and 1 denote the absence and the presence of an atom). To obtain the atom pattern, we considered a binarized version of the coefficient matrix ϴ, using a threshold that allowed us to discard the smallest noisy coefficients due to the inexactness of the minimization algorithm.

The algorithm then inferred a mixture M of trees T_*k*_,: $M = {\displaystyle {\sum}_{\boldsymbol{k}=1}^{\boldsymbol{K}}}{a}_{\boldsymbol{k}}{T}_{\boldsymbol{k}},$ assuming that the data are generated by more than one stochastic process. Each tree of the mixture represents the probability distribution over the 2^J^ possible patterns (with J atoms) associated with a set of events, and it is defined by vertices (random binary variables showing the occurrence of a single event) and weights (representing the conditional probability between events). The sum of weights *a*_***k***_ associated with each tree sums to 1, including the random mutations tree, a *noisy* tree associated with random mutations and represented by a star (all atoms belong to the first level and have a distance of one to the root node). Given a mixture of trees M and an atom pattern associated with a sample x, one can estimate how well the mixture represents such a pattern by evaluating the likelihood of the pattern for each tree in the mixture: $L\left(x\Big|M\right) = {\displaystyle {\sum}_{k=1}^K}L\left(x\Big|{T}_k\right)$. The likelihood *L*(*x*|*T*_*k*_) indicates how likely it is that the sample *x* belongs to the probability distribution defined by *T*_*k*_. It must be noted that we used MTreeMix differently from the method for which it was originally proposed. In particular, the approach was designed for inferring the phylogenetic trees of single mutations. In other words, the algorithm can work on lists of single mutations. In our case, we used the same approach to infer a hierarchical structure (tree) of patterns of mutations, *i.e.,* the atoms identified by E-FLLat. Our model indicates that stage 4 NB tumors show more complex CNA compared to the 4S genetic tree. This is clearly indicated by the necessity to represent the oncogenic tree of stage 4 tumors using 42 atoms (Additional file 2 Figure S2). Additionally, GIP model requires two different oncogenic trees for stage 4 tumors compared to the one identified for stage 4S tumors; in both cases, the level of noise (associated with the random mutations tree) is comparable (0.3 for stage 4S and 0.37 for stage 4), However both stage 4 oncogenetic trees are marked diverged, up to five sublevels, explaining the greater variability and complexity of stage 4 NBs, also indicating their greater malignancy.

The increase of numerical CNAs observed in 4S tumors, supports the chromosome endomitosis and abnormal mitosis as triggering events for malignant transformation. The endomitosis process [31] has been observed in a variety of physiological processes such as during the embryonic development, where the cells, as a nutriment and protection of the embryo, skip the cytokinesis step resulting in an increase of the ploidy [36]. The endomitosis process has been also described in cancer cells [37]. Kaneko and Knudson [38] reported the occurrence of endomitosis in stage 4S cells. This process can generate aneuploid cells that undergoing to clonal expansion contribute to stage 4S tumor development. Because the majority of 4S tumors regress spontaneously, in the GIP model we may speculate that 4S tumor cells, upon mitotic catastrophe, could undergo to apoptotic program to eliminate themselves, as initially proposed [39]. Another hypothesis already described in the stage 4S regression [40] indicates that the same polyploidy cells could differentiate and then regress. The 4S differentiation/regression process is further supported by our previous observation concerning upregulation of several genes belonging morphogenesis and differentiation, in particular the stage 4S overexpressed genes implicated in peripheral nervous system development and in Ras-mediated cell death programs [8]. The endoreplication can further generate high DNA instability, rendering the genome prone to structural chromosome damage [41]. This situation leads to the co-existence, inside the tumor cells, of both numerical and structural CNAs, a condition often observed in stage 4 [6, 8]. Contrarily to stage 4S, the stage 4 cells accumulate several genetic aberrations conferring proliferative advantage and capacity to circumvent programmed cell death [6]. Additionally, since in this high aggressive tumor, the 17q gain is the most common aberrations as the initial GIP event, we could also hypothesize that this genomic imbalance represents the starting event that force to the tumorigenesis of stage 4 NBs.

The structural chromosome gain often results in a consequently overexpression of genes located within these regions. Specifically several genes, mapping in the long arm of chromosome 17, have been found overexpressed in NB. One of the most important gene within this region, is *Survivin* (*BIRC5*) (17q25), that encodes for an antiaptototic protein [42]. Survivin expression has been found markedly upregulated in neuroblastomas, and high level of expression also correlated with poor prognosis [43, 44]. Similarity NME/NM23 nucleoside diphosphate kinase 1 (*NME1*) mapping in 17q21.3, has been also found overexpressed in some NBs and the upregulation correlated with metastatic disease [45].

It is interesting to note that in GIP model loss of chromosomes occur in the late event of carcinogenesis. In particular, stage 4S tumors show chromosome deletion at different sub-levels of the atoms’ tree (second A5, A6; third: A4, A9, A13 forth A8, A11). Notably, in stage 4S genetic tree, only 1 out of 6 nodes displays diverse sublevels, with deletions mainly affecting the short arm of chromosome 14. Loss of 14q has been already involved in the NB initiation/progression [46] and may explain why a small portion of 4S NBs develops toward a more aggressive disease.

On the other hand, the stage 4 tree shows loss at 11q that is not observed in stage 4S tumors, and this deletion was present either in first (stage 4, left tree) and in the second level (stage 4, right tree). Interestingly in both stage 4 trees, 11q deletion is occurring before 3p loss. This observation underlines the critical role of 11q deletion in stage 4 tumors suggesting that structural 11q aberration confers tumor aggressiveness as consequence of chromosome instability. Additionally, we also observe 1p deletion at the secondary levels of stage 4 trees. Both these regions have been associated with an unfavorable clinical outcome and older patients [47]. All above data are in agreement with Kaneko and Knudson [38] that suggested how chromosome loss may occur after endoreplication and demonstrate that chromosome deletion is a late event linked to higher aggressiveness.

Differently from chromosome gain, the chromosome loss may produce breakage of gene sequence and dramatically lack of the gene function. Chromosome 1p36 deletion has been observed in approximately 36 % of primary tumors and several studies indicate this region containing more than one NB suppressor genes including Tumor Protein p73 (*TP73)* (1p36.32) [48], cyclin-dependent kinase 11B (*CDK11B*) (1p36.33) [49], neuroblastoma 1, DAN family BMP antagonis*t (NBL1)* (1p36.13) [50] and paired box 7 (*Pax7)* (1p36.13) [51]. In addition to chromosome 1p other regions such as 3p, 11q and 14q, frequently lost in NB, were also identified to contain putative NB suppressor genes. Specifically, RAS-association domain family 1 isoform A and (*RASSF1A*), mapping in 3p21.3, is a pro-apototic RAS effector and plays a key role in the DNA repair [52]. Cell adhesion molecule 1 (*CADM1)* gene, involved in the cell junction organization, was also reported as a good candidate NB suppressor gene that can be damaged by the deletion of the 11q23 region [53]. Lastly, chromosome 14q23 deletion, was observed in about 22 % of NB and this locus contains MYC associated factor X (*MAX*) (14q23.3), that gives dimerization with *MYCN* gene [54], one of the most important oncogene associated with NB aggressiveness [55]. Lack of *MAX* repression function by chromosome 14q gene deletion may allow the overexpression of *MYCN* gene, as reported in aggressive NB. In conclusion, as suggested by GIP model, the occurring of chromosome 1p, 3p, 11q, and 14q deletions might increasing the aggressiveness of the tumor by damaging important NB suppressor genes.

Finally, because previous reports [7, 8] indicate that chromosome damages accumulates with increasing patient age, we evaluated the correlation between CNAs and patient age. We found a significant (*p =* 0.025) association between patient age and chromosome loss exclusively for stage 4 (atoms A15, A9; data not shown), supporting the hypothesis that 3p and 11q chromosome deletion accumulates in older patients.

## Conclusions

**Fig. 5:**
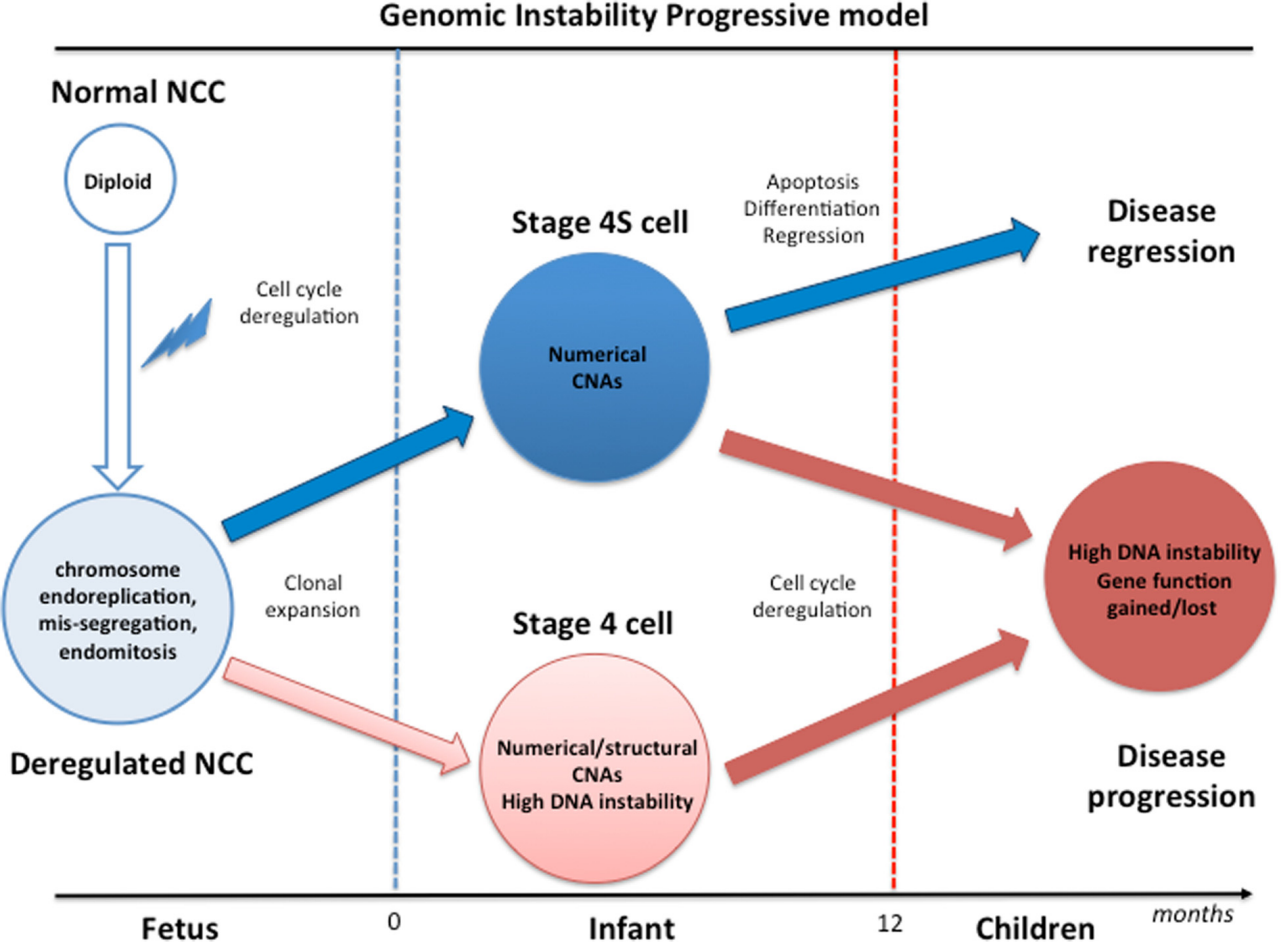
Schematic representation of the Genome Instability Progressive model of metastatic Neuroblastoma. The GIP model suggests a common ancestor for metastatic “stage 4S and 4 NBs”, showing that a deregulated endomitosis, chromosome mis-segregation and abnormal mitosis in the neural crest progenitors lead to aneuploid cells. This cell may generate “Stage 4S cell clone”, characterized mainly by numerical aberrations. However the clone maintains the capacity to active cell death or differentiation programs as mechanism to escape the catastrophic mitosis. Conversely the deregulated neural crest cells (NNC) may also generate “Stage 4 cell clone” with high genomic instability resulting in complex chromosome rearrangements. Finally, in the GIP model chromosomal deletions are late events, resulting in increasing of genomic chaos and progressive increase of genomic instability, with consequent tumor progression

In conclusion, we propose for the first time a model of carcinogenesis for metastatic NB based on dictionary learning. Our model suggests that an aberrant regulation of the endomitosis could correlate to carcinogenesis process of metastatic NB. Afterwards, the polyploidy cells evolve in malignant clonal cell expansion generating stage 4S or stage 4 NB, each of which is characterized by distinct genomic features. The former disease characterized by an increase of numerical chromosome aberrations, is able to activate a cell death or differentiation programs to escape the catastrophic mitotic. Conversely, stage 4 NBs show several complex chromosome rearrangements, where the chromosome deletions occur as late event, resulting in an increase of the genomic chaos and a progressive increase of chromosome instability, with consequent rapid disease progression (Fig. 5).

Future investigations of the NB carcinogenesis process can address future therapies to re-regulate the malignant cell cycle.

## Additional files

Additional file 1: Figure S1. PCA analysis for stage 4S (top) and stage 4 (bottom) tumors. The vertical lines show the numbers of eigenvectors needed to explain 50 %, 70 % and 90 % of the variance in the data. (TIFF 1811 kb) Additional file 2: Figure S2. A plot of the stage 4 samples (top) and the corresponding reconstruction of the stage 4 sample data using J = 42 atoms (bottom). Red color indicates a gain, whereas blue corresponds to loss, as shown by the color bar. (TIFF 1521 kb)

## Abbreviations

aCGH: Array comparative genomic hybridization
CNAs: Copy number aberrations
DL: Dictionary learning
E-FLLat: Enhanced fused lasso latent feature Model
GEO: Gene expression omnibus
GIP: Genome instability progressive
HG19: Human genome
NB: Neuroblastoma
PCA: Principal component analysis
QC: Quality control
TV: Total variation
UCSC: University of California, Santa Cruz (on-line genome browser)

## Footnotes

1 http://hgdownload.soe.ucsc.edu/goldenPath/hg19/database/

2 http://genome.ucsc.edu/cgi-bin/hgLiftOver.
