# Supplementary figures and images for: Genome instability model of metastatic neuroblastoma tumorigenesis by a dictionary learning algorithm
Source: BMC Med Genomics. 2015 Sep 10;8:57. doi: 10.1186/s12920-015-0132-y (PMC4566396; doi:10.1186/s12920-015-0132-y)

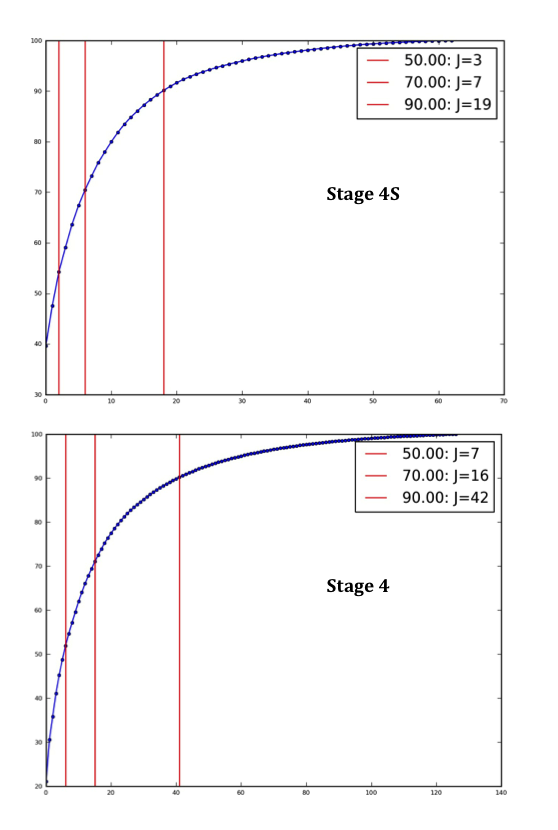

Supplement: Additional file 1: Figure S1. — PCA analysis for stage 4S (top) and stage 4 (bottom) tumors. The vertical lines show the numbers of eigenvectors needed to explain 50 %, 70 % and 90 % of the variance in the data. (TIFF 1811 kb) [file 12920_2015_132_MOESM1_ESM.tiff]

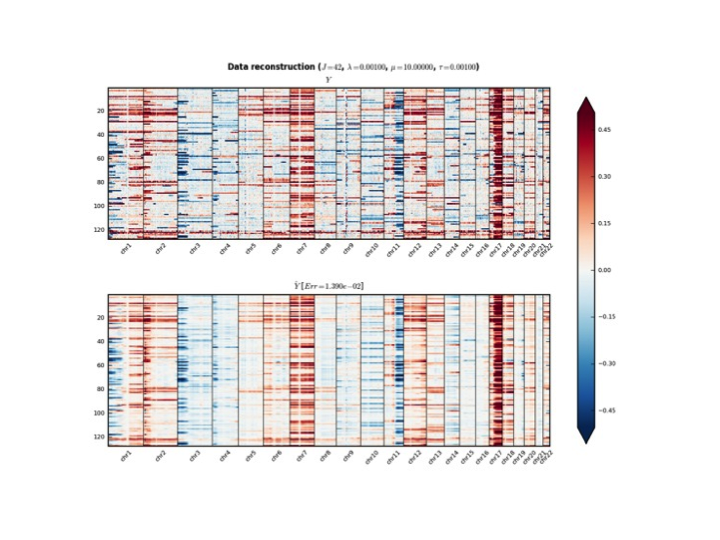

Supplement: Additional file 2: Figure S2. — A plot of the stage 4 samples (top) and the corresponding reconstruction of the stage 4 sample data using J = 42 atoms (bottom). Red color indicates a gain, whereas blue corresponds to loss, as shown by the color bar. (TIFF 1521 kb) [file 12920_2015_132_MOESM2_ESM.tiff]
